# Supplementary material for: The Systems Biology Research Tool: evolvable open-source software
Source: BMC Syst Biol. 2008 Jun 29;2:55. doi: 10.1186/1752-0509-2-55 (PMC2446383; doi:10.1186/1752-0509-2-55)
Supplement: Additional file 1 — SBRT Archive. An archive of the current version of the Systems Biology Research Tool. [file 1752-0509-2-55-S1.zip › sbrt-1.4.0/doc/developers_guide/api/sbrt/kernel/util/UnmodifiableIterator.html]

UnmodifiableIterator


|  |  |  |
| --- | --- | --- |
| **Overview** \| **Package** \| **Class** \| **Use** \| **Tree** \| **Deprecated** \| **Index** \| **Help** | |  |
| **PREV CLASS**   **NEXT CLASS** | **FRAMES**    **NO FRAMES**     **All Classes** |  |
| SUMMARY: NESTED \| FIELD \| CONSTR \| METHOD | DETAIL: FIELD \| CONSTR \| METHOD |  |


---


## sbrt.kernel.util Class UnmodifiableIterator<E>

```
java.lang.Object
  sbrt.kernel.util.UnmodifiableIterator<E>
```

**Type Parameters:**: `E` - the element type.

**All Implemented Interfaces:**: java.util.Iterator<E>

---

``` public class UnmodifiableIterator<E> extends java.lang.Object implements java.util.Iterator<E> ```

This class is used to create an iterator that cannot modify its
underlying collection.
If a method returns an `UnmodifiableIterator`, it's clear that the
underlying collection cannot be modified with that iterator and that
`UnsupportedOperationException` will be thrown
if `remove()` is called.

**Author:**
:   This class was written and documented by Jeremiah Wright while
    in the Wagner lab.

---

**Constructor Summary**

`UnmodifiableIterator(java.util.Iterator<E> i)`
  
          Constructs a new unmodifiable iterator from the provided iterator.


| **Method Summary** | |
| --- | --- |
| `boolean` | `hasNext()`             See Iterator.hasNext(). |
| `E` | `next()`             See Iterator.next(). |
| `void` | `remove()`             Throws `UnsupportedOperationException`. |

**Methods inherited from class java.lang.Object**

`clone, equals, finalize, getClass, hashCode, notify, notifyAll, toString, wait, wait, wait`

**Constructor Detail**

### UnmodifiableIterator

```
public UnmodifiableIterator(java.util.Iterator<E> i)
```

:   Constructs a new unmodifiable iterator from the provided iterator.

    **Parameters:**: `i` - the iterator for which an unmodifiable version is created.


**Method Detail**

### next

```
public E next()
```

:   See Iterator.next().

    :   **Specified by:**: `next` in interface `java.util.Iterator<E>`

---


### hasNext

```
public boolean hasNext()
```

:   See Iterator.hasNext().

    :   **Specified by:**: `hasNext` in interface `java.util.Iterator<E>`

---


### remove

```
public void remove()
```

:   Throws `UnsupportedOperationException`.

    :   **Specified by:**: `remove` in interface `java.util.Iterator<E>`
    :   **Throws:**: `java.lang.UnsupportedOperationException`


---


|  |  |  |
| --- | --- | --- |
| **Overview** \| **Package** \| **Class** \| **Use** \| **Tree** \| **Deprecated** \| **Index** \| **Help** | |  |
| **PREV CLASS**   **NEXT CLASS** | **FRAMES**    **NO FRAMES**     **All Classes** |  |
| SUMMARY: NESTED \| FIELD \| CONSTR \| METHOD | DETAIL: FIELD \| CONSTR \| METHOD |  |


---
